# DNA-conjugated gold nanoparticles based colorimetric assay to assess helicase activity: a novel route to screen potential helicase inhibitors

**DOI:** 10.1038/srep44358

**Published:** 2017-03-13

**Authors:** Jashmini Deka, Aditya Mojumdar, Pietro Parisse, Silvia Onesti, Loredana Casalis

**Affiliations:** 1 Nano Innovation Laboratory, Elettra-Sincrotrone Trieste S.C.p.A., Trieste, Italy; 2 Structural Biology Laboratory, Elettra-Sincrotrone Trieste S.C.p.A., Trieste, Italy

## Abstract

Helicase are essential enzymes which are widespread in all life-forms. Due to their central role in nucleic acid metabolism, they are emerging as important targets for anti-viral, antibacterial and anti-cancer drugs. The development of easy, cheap, fast and robust biochemical assays to measure helicase activity, overcoming the limitations of the current methods, is a pre-requisite for the discovery of helicase inhibitors through high-throughput screenings. We have developed a method which exploits the optical properties of DNA-conjugated gold nanoparticles (AuNP) and meets the required criteria. The method was tested with the catalytic domain of the human RecQ4 helicase and compared with a conventional FRET-based assay. The AuNP-based assay produced similar results but is simpler, more robust and cheaper than FRET. Therefore, our nanotechnology-based platform shows the potential to provide a useful alternative to the existing conventional methods for following helicase activity and to screen small-molecule libraries as potential helicase inhibitors.

Helicases are ubiquitous enzymes, found in viruses, bacterial, archaeal and eukaryotic cells. They act as motor proteins to separate or remodel DNA or RNA substrates, using ATP as energy source; their activity is essential in nucleic acid metabolism, playing a key role in a variety of processes, such as DNA replication, repair, recombination, translation, RNA transport, etc. Not surprisingly, given their essential tasks in living organisms, they are emerging as an important class of targets for antiviral, antibiotic and anti-cancer drugs^1^,^2^. As an example of the latter, helicases (and in particular RecQ helicases) are essential to overcome the effect of chemotherapeutic drugs that damage DNA, making them attractive targets for inhibitors to make cancer cells more sensitive to chemotherapy^3^.

The search for specific inhibitors of helicases strongly relies on the development of easy, cheap, fast, reproducible biochemical assays, suitable for high-throughput (HT) screening. Currently used methods include both ATPase and helicase assays. ATP hydrolysis methods rely on the colorimetric detection of the phosphate^4^,^5^ or the detection of ADP through antibody-based ADP sensors or ADP-coupled reactions^6^. Nucleic acid unwinding assays are typically based on radioactive or fluorescent substrates^7^. Colorimetric ATPase assays are generally simpler, less expensive and more suited for HT screenings, but can miss inhibitors that abolish DNA/RNA unwinding without affecting ATP hydrolysis. On the other hand, strand separation assays are more complex and tend to require expensive and sophisticated reagents, such as labelled oligonucleotides^8^. Among them, the ones that can more easily be adapted for HT screens are those based on fluorescent resonance energy transfer (FRET), but are subjected to some drawbacks, including high costs, poor stability of the substrate and compound interference^2^,^9^,^10^,^11^. The latter is particularly serious, as compound libraries include many molecules that absorb or emit light at wavelengths that overlap with those of the fluorophores being monitored^2^. There is therefore the need to explore alternative methods to measure helicase activity.

Here we report a novel method for the measurement of helicase activity, based on the properties of functionalized gold nanoparticles (AuNPs). The ease of synthesis/functionalization and unique optical properties of AuNPs allow for their use in the development of newer diagnostic methods which are safer and easier than the conventional existing methods. A number of reports exist on the use of AuNP based structures to detect and measure the activities of many enzymes belonging to various classes such as hydrolases, transferases, oxidoreductases etc.^12^,^13^,^14^. Colorimetric techniques either follow the aggregation of AuNPs or the disintegration of AuNP aggregates, in response to the enzyme activity. In the present work, we have constructed nanoparticles conjugated to a specifically-designed DNA substrate, so as to monitor the DNA unwinding properties of a helicase. The current set-up is designed for a helicase with a 3′–5′ directionality, but it can easily be adapted for 5′–3′ helicases.

As a test case, we used the human RecQ4 helicase. RecQ helicases are ubiquitous nucleic acid unwinding enzymes, playing a vital role in maintaining genomic stability by acting at the interface of replication, repair and recombination. They are involved in DNA repair, homologous recombination, telomere maintenance, mitochondrial genome maintenance and DNA replication^15^,^16^,^17^. Three out of five human RecQ helicases are associated to genetic disorders, characterized by genomic instability, premature aging and predisposition to cancer^18^. Mutations in RecQ4 are associated to Rothmund-Thomson Syndrome (RTS), RAPADILINO and Baller-Gerold Syndrome^19^,^20^. Although RecQ4 deficient RTS patients have an elevated risk of developing osteosarcoma, overexpression of RecQ4 has been reported in human osteosarcoma, prostate and breast tumour samples^21^,^22^. The human RecQ4 helicase consists of 1208 amino-acids, including a conserved helicase core^23^,^24^. In most RecQ helicases the catalytic core is followed by a RecQ-C-terminal (RQC) domain, that has been proposed to have a crucial role in the helicase activity, by providing an aromatic residue acting as essential “pin” that physically disrupts the dsDNA base-pairing^25^,^26^; a bioinformatic analysis recently suggested the presence of a non-canonical RQC domain in RecQ4^27^. Despite the role of RecQ4 in genetic disorders and carcinogenesis, not much information is available about its mechanism of action.

By using a novel AuNP helicase assay method, the catalytic core of human RecQ4 has been characterized and the results compare favourably with those obtained with standard FRET-based methods. A number of site-directed mutants have been analysed, confirming the importance of key residues in the putative RQC domain for the helicase activity.

## Results and Discussion

### Substrate preparation

To obtain the substrate for monitoring helicase activity, we prepared DNA-functionalized AuNPs which aggregate due to the annealing of the complementary DNA strands^28^. The assay relies on the disruption of the aggregates and the consequent solubilization of the particles upon DNA unwinding. The course of the reaction can be simply followed by monitoring the surface plasmon resonance (SPR) absorption spectra of AuNPs using a spectrophotometer. Surface plasmon is the collective oscillations of the free electrons on the surface of nanoparticles which is in resonance with the frequency of the visible light interacting with them. For 20 nm sized monodispersed AuNPs, the SPR phenomenon causes the absorption of light at about 524 nm^29^. The absorption peak shifts to longer wavelengths as the AuNPs aggregate. This shift in the absorbance can be used to monitor the change in the aggregation state of the AuNPs, due to the DNA unwinding catalysed by a helicase.

**Figure 1:**
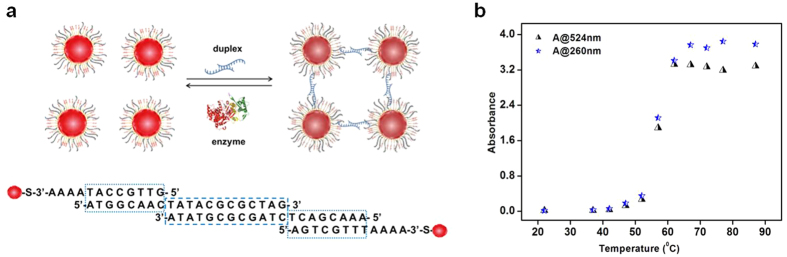
(**a**) Schematic diagram illustrating the formation of the DNA/TOEG6@AuNPs aggregates. A mixture of two batches of nanoparticles with DNA1 and DNA2, respectively, is mixed with a DNA duplex with sticky ends partially complementary to DNA1 and DNA2 (Dup-DNA1/Dup-DNA2), which triggers the aggregation. DNA unwinding by a helicase reverses the aggregation. (**b**) Melting curves of the substrate as monitored by the absorbance at 524 nm and 260 nm.

The protocol for preparing the substrate has been previously described^30^. Briefly, we used 20 nm sized AuNPs functionalized with a mixed self-assembled monolayer (SAM) of DNA and (1-mercaptoundec-11-yl)hexa(ethylene glycol) referred to as TOEG6; (DNA/TOEG6@AuNP). The function of TOEG6 in the SAM is to provide salt-stability and specificity to the DNA conjugated AuNPs. The preparation of the substrate for the enzymatic assay was achieved by mixing two batches of DNA/TOEG6@AuNP (with DNA1 and DNA2 respectively, Table 1) in the presence of a duplex which has DNA strands partially complementary to the DNA strands on each batch of DNA/TOEG6@AuNP. The formation of DNA/TOEG6@AuNP based aggregates is schematically shown in Fig. 1a.

Melting experiments with the assembled substrates were carried out to confirm that the aggregate formation was based on DNA-DNA recognition and not due to non-specific interactions. For this, the aggregate was subjected to heating with a linear increase in temperature from 22 to 87 °C while recording the spectrum of the solution, between 200 nm and 800 nm in a UV-vis spectrophotometer. The absorbance was measured at 524 nm (corresponding to the DNA/TOEG6@AuNP) and at 260 nm (corresponding to the DNA), and plotted against the temperature (Fig. 1b), showing a sharp transition in the absorbance at about 60 °C. This is in agreement with previous studies on the melting patterns of DNA@AuNP based aggregates^31^. It was observed that the melting curves of DNA tagged with AuNPs are sharper than that of DNA alone. As expected, the absorbance variation at 260 and 524 nm followed the same pattern, since heating leads to the disintegration of the aggregates, increasing the amount of DNA as well as separated nanoparticles in the solution. A more detailed analysis on the formation of the aggregate based on DNA recognition and annealing were reported in our previous work^30^. All studies supported the fact that the formation of the aggregate was guided by the DNA binding specificity and that it could serve as the substrate to follow DNA unwinding by a helicase.

### Helicase assay

**Figure 2:**
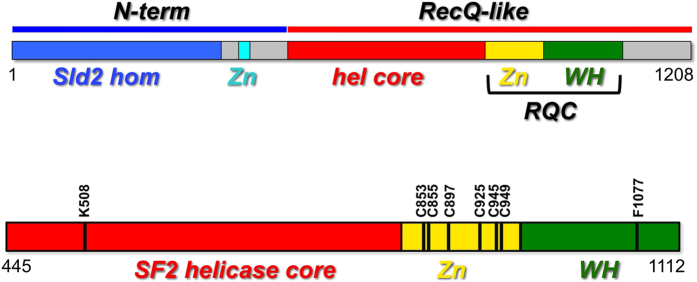
A schematic diagram of the domain organization of human RecQ4, showing the N-terminal region (blue) followed by helicase core (red) and the putative RQC domain (consisting of a Zn-binding domain, in yellow, and a winged helix domain, in green). In the lower panel is shown the catalytic core used in this study comprising of the helicase and RQC domain. The site-directed mutants analysed in this study are shown.

As RecQ4 includes a long, partially unstructured N-terminal domain, which is apparently not involved in the helicase activity^32^, the enzyme used in the assay corresponds to a deletion mutant, which includes the helicase core and the putative RQC domain of the human RecQ4 helicase (Fig. 2). The protein was expressed and purified to homogeneity in bacterial cells^33^.

**Figure 3:**
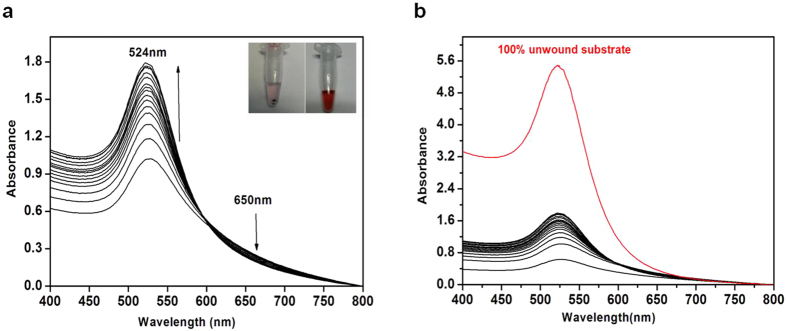
(**a**) Surface Plasmon Resonance curves of the reaction mixture (helicase buffer with substrate, enzyme and ATP) as a function of time. The inset shows the colour of the substrate before and after enzyme addition. (**b**) The SPR graph curve of the 100% unwound substrate as compared with the curves obtained as a result of the enzymatic reaction.

To monitor the RecQ4-catalysed enzymatic reaction, helicase assay buffer was added to the AuNP aggregate (hereafter referred to as substrate) that was then transferred to a Perkin Elmer UV-vis spectrophotometer cuvette, followed by the addition of the enzyme and ATP. The addition of the enzyme in the presence of ATP immediately causes a dramatic colour change: the solution turns into a purple suspension, followed by a slower colour variation towards red (Fig. 3a); this is caused by the disruption of the DNA-linked aggregates and the consequent solubilisation of the nanoparticles. To quantitatively monitor these events, the time dependent UV-vis spectra of the solution were recorded. The time dependent UV-vis graphs for the experiment, with an enzyme concentration of 60 nM, are shown in Fig. 3. It can be observed that with increasing time, the absorbance spectra of the reaction showed an increase in the maximum absorbance at 524 nm and a decrease at 650 nm. This is due to RecQ4 unwinding the double-stranded DNA, breaking the substrate into smaller parts and consequently releasing the DNA/TOEG6@AuNPs in the solution, which absorbs in the visible region of the electromagnetic spectrum. The shoulder at 650 nm corresponds to the assembled state of the nanoparticles and indeed decreases as the 524 nm peak (corresponding to the monodispersed particles in solution) increases.

To derive quantitative data from these changes in the SPR spectra, we considered the ratio A@524 nm:A@650 nm at each time interval and converted it into the percentage of unwinding using the following relation:


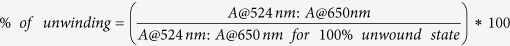


In the above relation, the ratio for 100% unwound state corresponds to the value for the substrate DNA/TOEG6@AuNP boiled at 95 °C for 10 minutes (red curve in Fig. 3b).

**Figure 4:**
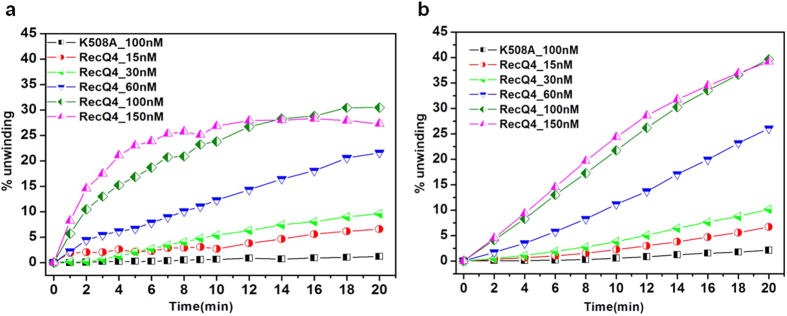
(**a**) DNA unwinding as a function of time for various concentrations of wild-type RecQ4 (from 15 to 150 nM) and 100 nM of WA mutant (K508A), obtained from the AuNP-based method. (**b**) DNA unwinding as a function of time for various concentrations of RecQ4 and WA mutant (K508A) obtained from a FRET-based method. All the experiments were done in triplicate and the error bars are not shown for the sake of clarity.

The analyses were performed for five different concentrations of the enzyme, corresponding to 15 nM, 30 nM, 60 nM, 100 nM and 150 nM respectively. The percentage of unwinding against the respective time interval for various concentrations of the enzyme is shown in Fig. 4a. As a control we used a protein mutant (WA) where the essential lysine 508 (the key residue within the Walker A motif, involved in ATP binding) has been mutated to an alanine.

As evident from the data, the extent of unwinding increases with time and is dependent on the concentration of the enzyme. The plots corresponding to higher enzyme concentrations (100 nM and 150 nM) initially show a relatively steep increase in unwinding with a tendency to reach a plateau (around 15–20 mins) compared to the plots corresponding to the lower enzyme concentrations (15 nM, 30 nM and 60 nM) where the value rises slowly and doesn’t reach any plateau after 20 minutes of reaction time. As expected, the Walker A mutant (K508A) displays no detectable unwinding activity showing that the disaggregation of the substrate is specifically due to the RecQ4 unwinding activity and is not an artefact.

To explain the presence of the plateau, it is worth mentioning that beside the ATP-dependent DNA unwinding activity, RecQ4 can also catalyse DNA annealing in an ATP-independent manner^33^,^34^. The annealing reaction may thus compete with the DNA unwinding reaction, so as to reach equilibrium. In the present case the maximum % of unwinding observed for 100 nM and 150 nM enzyme is 25% to 30%.

In order to validate the flexibility of this method, we prepared two additional substrates: a longer blunt-ended duplex and a substrate with a 3′ tail (Supplementary Figure S1). The enzyme was able to unwind both substrates. These results confirm that the AuNPs-based assay is flexible enough to accommodate a variety of nucleic acid substrates.

### Comparison with a FRET-based assay

**Figure 5:**
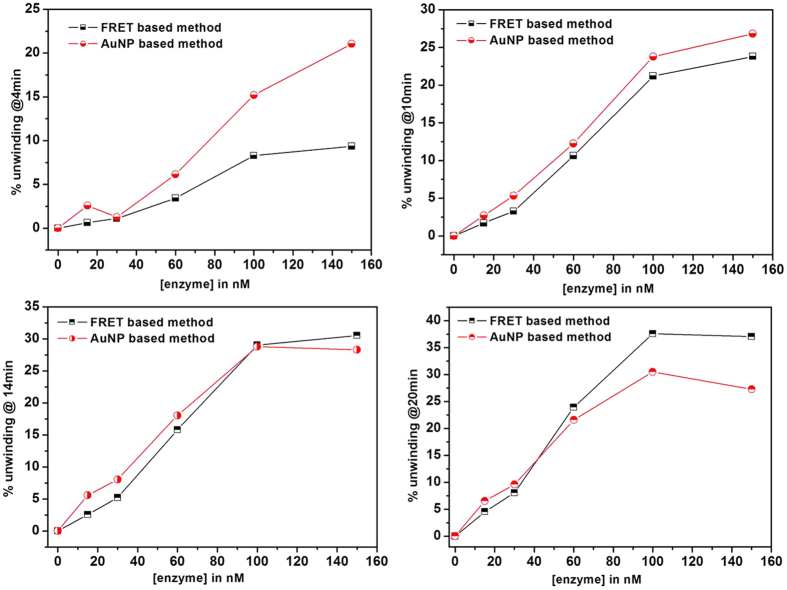
DNA unwinding for various concentrations of wild-type RecQ4 (from 0 to 150 nM) at 4, 10, 14 and 20 minutes of reaction. All the experiments were done in triplicate and the error bars are not shown for the sake of clarity.

As a control, DNA unwinding experiments in similar conditions were performed using a conventional FRET based assay. FRET data (Fig. 4b) show that the unwinding is slow and keeps increasing with time without reaching a plateau within the time range used for the AuNP-based assay. The comparative data of percentage unwinding at 4^th^, 10^th^, 14^th^ and 20^th^ minutes respectively (as obtained by the two methods) for various concentrations of the enzyme are shown in Fig. 5.

When the unwinding efficiency of the two methods is compared at 4, 10, 14 and 20 minutes (Fig. 5), for various protein concentration, there is a good match at 10 and 14 minutes, with some discrepancies at lower and higher time points. At the lower end (4 min) a higher activity is measured in the AuNP-based assay, whereas at 20 min the trend is reversed, with the FRET methods providing a higher estimate of the activity. A possible explanation of this behaviour is that the enzyme accumulates at/around the aggregated DNA-AuNPs, as described for protein-corona^35^,^36^, and once ATP is added, a rapid unwinding activity is observed while in the FRET-based assay the double-stranded DNA substrates are homogenously dispersed in the whole reaction volume, leading to a slow initial unwinding. Similar observations of increase in enzymatic activity in the presence of citrate stabilized AuNPs have been previously reported^37^. Within 10 to 14 minutes the enzyme in both the systems might get access to equivalent amount of the available double-stranded DNA substrates and thus shows a similar unwinding activity. By 20 min reaction time the enzyme in the FRET assay continues to unwind the soluble substrates, while in the AuNP based assay, the availability of multiple DNA strands per AuNP may favour re-aggregation due to strand annealing.

Despite these small differences in enzyme behaviour observed at the initiation of the reaction, this novel AuNP based colorimetric system provides a good alternative to other existing methods to detect the unwinding activity of a helicase and to quickly obtain comparative unwinding data. In contrast to FRET, our method does not require any lengthy and costly labelling step or specific storage conditions. Instead, our approach involves easy and fast steps for the preparation of the DNA-AuNP based substrate, which is stable in terms of time and buffer conditions (e.g. ionic strength, pH, etc.) and a very simple instrument like UV-vis spectrophotometer for the readout, making it competitive over the fluorescence based assay.

### Analysis of site-directed mutants

**Figure 6:**
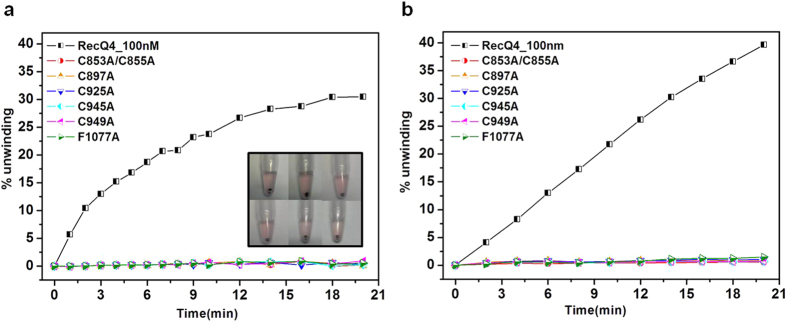
DNA unwinding as a function of time for various mutant proteins (at a concentration of 100 nM), as obtained from (**a**) AuNP and (**b**) FRET based method. The inset in (A) shows the colour of the substrate after 20 minutes. All the experiments were done in triplicate and the error bars are not shown for the sake of clarity.

In order to further test the capabilities of the methods for biochemical characterization, we tested several site-directed mutants of RecQ4. The amino acid residues shown to play a role in enzyme function^33^, were mutated to alanine (shown in Fig. 2). The residues include the cysteines identified as putative Zn^2+^ ligands (C853, C855, C897, C925, C945 and C949) and the phenylalanine (F1077) which is predicted to act as a pin. Various mutant proteins (C853A/C855A, C897A, C925A, C945A, C949A and F1077A) were expressed and purified, and DNA unwinding was then tested at a protein concentration of 100 nM. The time-dependent course of the unwinding reaction is shown for all the six mutants (Fig. 6a). All mutants lost the ability to unwind the substrate and thus disrupt the AuNP aggregates, as confirmed by the FRET assay (Fig. 6b). This indicates an essential role of all the cysteine residues in the putative Zn-binding domain and the phenylalanine at the tip of the predicted WH β-hairpin in DNA unwinding, hence validating the predictions made by the *in-silico* analysis^27^, providing important insights on the biochemistry and architecture of the protein.

## Conclusions

We have developed a unique DNA-conjugated AuNP based method to detect and monitor the unwinding activity of DNA helicases, and we tested it by characterising the activity of the catalytic core of human RecQ4 helicase and site-directed mutants. The method is guided by the changes in the optical properties of the AuNP in the DNA–AuNP aggregated based substrate, caused by the DNA unwinding activity of the enzyme.

This is the first time that such a simple, easy, robust and cheap nanoparticle-based method for following the activity of a DNA helicase has been proposed. The method was validated by comparison with a conventional fluorescence-based assay and the results from the two methods were found to be in good agreement. In addition to the ease of preparation and stability of the substrate, our method provides a naked eye test for a comparative activity assay of the enzyme as well as its mutants, and can also be used to quantitatively determine enzymatic activity. The method was tested on a monomeric helicase belonging to the SF2 superfamily, with a 3′-5′ directionality. We expect the method to be less suitable for hexameric helicases (Superfamilies SF3-SF6) as their bulky structures may sterically interferes with the nanoparticles. However, we anticipate it to be easily adaptable to SF1/SF2 5′-3′ helicases, with the design and optimization of appropriate AuNP-DNA conjugates and DNA linkers. As superfamilies SF1/SF2 constitute by far the largest group of helicases, the methods is potentially adaptable to most enzymes of pharmacological interest. Further optimisation will make this method well-suited for high-throughput commercial screenings and robotic manipulations. Its simplicity, easy detection and robustness make it a competitive alternative to the conventional methods to assay helicase activity and to quickly and easily test the activity of small molecule inhibitors against helicases.

## Methods

### Preparation of citrate stabilized AuNPs (cit@AuNPs)

A detailed protocol for DNA-conjugated AuNPs preparation is reported in Deka *et al*.^30^. Briefly cit@AuNPs were prepared by a known method of citrate reduction of HAuCl_4_. A 1.1 ml aliquote of a 1.73 × 10^−2^ M HAuCl_4_ solution (Sigma-Aldrich Chemical Co.) was added to 43.6 ml of MilliQ grade water in a volumetric flask and brought to boil on a Bunsen burner. Then 300 μl of 72 mg/ml trisodium citrate 2-hydrate (Merck) was added and the boiling was continued for another 30 min to ensure complete reduction of HAuCl_4_. The solution turned from colorless to deep red, indicating the synthesis of AuNPs. The red solution was removed from the heat and allowed to cool down at room temperature. Freshly prepared AuNPs were used for every experiment. The diameter of the cit@AuNPs obtained was determined to be 19 ± 2 nm from Scanning Electron Microscopy (SEM) analysis.

### Synthesis of DNA/TOEG6@AuNP

All the oligonucleotides used for substrate preparation (Table 1) were purchased from biomers.net and are listed in Table 1. 5.22 μl of 100 μM ssDNA (DNA1 or DNA2) was added to 750.0 μl of AuNPs mixed by vortexing and left for 10 minutes. The pH of the mixture was subsequently adjusted to 4.3 by the addition of 1.5 ml of 0.1 M citrate-HCl buffer at pH 4.3. The mixture of ssDNA and AuNPs was incubated at room temperature for another 30 minutes after which 15.0 μL of 300.0 μM (1-mercaptoundec-11-yl)hexa(ethylene glycol) (TOEG6) (Sigma-Aldrich Chemical Co.) was added. The mixture was then vortexed well and left to rest for another 10 minutes. The solution was centrifuged in an eppendorf tube at a speed of 14,000 rpm for 10 minutes using a table top Beckman Coulter centrifuge and the supernatant was discarded to remove the unbound ssDNA and TOEG6 molecules from the functionalized AuNPs. The pellet was washed twice in 250 μl deionised water using the same centrifugation process as described above. The final pellet was resuspended in 250 μl of buffer A (10 mM sodium phosphate buffer of pH 7.0 and 1 M NaCl). Freshly prepared AuNPs were used for every experiment.

### Synthesis of AuNP-DNA aggregate

A duplex solution was prepared by mixing 5.22 μl of 100 μM each of dup-DNA1 and dup-DNA2 in 250.0 μl of buffer A (mentioned above), the duplex DNA was annealed by incubating the reaction mixture at 50 °C for 15 minutes followed by slow cooling. The solution thus prepared was added to the mixture of DNA1/TOEG6@AuNP and DNA2/TOEG6@AuNP. The solution turned purple in few minutes and settled down in few hours. The mixture was left at room temperature overnight. The precipitate (aggregate) obtained was separated from the colourless supernatant by centrifuging at 5,000 rpm for 10 minutes.

### Melting experiment with the AuNP-DNA aggregates

500 μl of Buffer A (mentioned above) was added to the AuNP-DNA aggregate in an eppendorf and kept in a water bath at 37 °C for 5 minutes. 50 μl of the mixture were pipetted into a cuvette for UV-vis spectrophotometry. AThe measurement was repeated after every 5 °C rising temperature till 87 °C. The A@260 nm (corresponding to DNA) and A@520 nm (corresponding to AuNP-DNA) were recorded and plotted against the respective temperatures.

### Activity assay of human RecQ4 helicase

The helicase assay was performed in buffer (20 mM Tris–HCl pH 7.5, 5 mM MgCl_2_, 50 mM KCl, 0.1 mg/ml BSA and 5% Glycerol), added to the AuNP-DNA substrates, to a final volume of 400 μl with 5 mM ATP and protein concentration ranging from 0 to 150 nM. Before starting the analyses, the instrument (Lambda 25 UV-Vis spectrometer, Perkin Elmer) was set to zero with the helicase assay buffer as the blank. The helicase activity of the enzyme was estimated by measuring the spectrum between 400 and 800 nm of the reaction in the cuvette.

### Cloning, expression and purification of recombinant RecQ4 protein

The nucleotide sequence encoding the helicase and RQC domain (residues 445 to 1112 of the human sequence) was PCR-amplified using the Platinum Pfx DNA polymerase (Invitrogen). The amplified fragment was subcloned into a pET-SUMO/CAT vector (Invitrogen) using the restriction free cloning method^38^. Site directed mutagenesis was carried out to mutate the lysine in the Walker A motif to alanine (WA: K508A) to be used as a control in the helicase assay. Several other mutations were produced (C853A/C855A, C897A, C925A, C945A, C949A and F1077A, Fig. 2). All the plasmids were sequence verified^33^.

The wild type and mutant proteins were expressed with a His-SUMO tag and were purified using Nickel affinity chromatography (HisTrap FF 5 ml column) with 50 mM Hepes pH 8.0, 0.5 M NaCl, 1 mM TCEP, 5% glycerol and 500 mM Imidazole buffer using a linear gradient. The tag of the purified protein was later cleaved using SUMO protease and was further purified using Heparin affinity chromatography (HiTrap Heparin 5 ml column) with 50 mM Hepes pH 7.5 and 1 M NaCl buffer using a linear gradient. Another step of purification was performed using a size exclusion chromatography (Superdex-200) with 20 mM Tris pH 8.0, 250 mM NaCl, 5% glycerol and 5 mM β-mercaptoethanol. Protein concentration was determined by measuring the absorbance at 280 nm, and protein purity was analyzed on SDS-PAGE. Purifications of all the mutant proteins were performed as described above for wild type protein^33^.

### Oligonucleotide preparation

The double stranded DNA substrates were prepared by annealing the complementary fluorescent labelled oligonucleotides in annealing buffer (10 mM Tris-HCl pH 7.5 and 50 mM NaCl) by heating at 95 °C for 8 min and slowly cooling to room temperature. The fluorescent labelled DNA oligonucleotides (6FAM label at 5′ end and BHQ1 label at 3′ end) are shown in Table 2.

### Fluorescence based helicase assay

The helicase activity was measured by using a fluorescence resonance energy transfer (FRET) based assay^7^. The assay was performed in helicase assay buffer (20 mM Tris–HCl pH 7.5, 5 mM MgCl_2_, 50 mM KCl, 0.1 mg/ml BSA and 5% Glycerol) with 10 nM Fork DNA substrate (F1:F2) and 5 mM ATP with protein concentration ranging from 0 to 150 nM. The fluorescence intensity was recorded using Infinite F200 PRO TECAN instrument. The reaction was incubated at 95 °C in the absence of enzyme to provide a measurement of 100% unwinding. The assay was done in triplicate.

## Additional Information

**How to cite this article**: Deka, J. *et al*. DNA-conjugated gold nanoparticles based colorimetric assay to assess helicase activity: a novel route to screen potential helicase inhibitors. *Sci. Rep.* **7**, 44358; doi: 10.1038/srep44358 (2017).

**Publisher's note:** Springer Nature remains neutral with regard to jurisdictional claims in published maps and institutional affiliations.

## Supplementary Material

Supplementary Information

## Figures and Tables

**Table 1:** Oligonucleotide sequences used for the AuNP assay.

Name	Oligonucleotide sequence
DNA1	5′-GTTGCCATAAAA-3′-SH
DNA2	5′-AGTCGTTTAAAA-3′-SH
Dup-DNA1	5′-ATGGCAACTATACGCGCTAG-3′
Dup-DNA2	5′-AAACGACTCTAGCGCGTATA-3′

DNA1 and DNA2 are thiol C3 modified at the 3′ end.

**Table 2:** Oligonucleotide sequences used for the FRET helicase assay.

Name	Sequence (5′-3′)
F1	[6FAM]CTACTACCCCCACCCTCACAACCTTTTTTTTTTTTTT
F2	TTTTTTTTTTTTTTGGTTGTGAGGGTGGGGGTAGTAG[BHQ1]

F1 is 6FAM labelled at 5′ end and F2 is BHQ1 labelled at 3′ end.
