# Supplementary material for: DNA-conjugated gold nanoparticles based colorimetric assay to assess helicase activity: a novel route to screen potential helicase inhibitors
Source: Sci Rep. 2017 Mar 13;7:44358. doi: 10.1038/srep44358 (PMC5347027; doi:10.1038/srep44358)
Supplement: Supplementary Information [file srep44358-s1.pdf]

DNA-conjugated gold nanoparticles based colorimetric assay to assess helicase activity: a novel route to screen potential helicase inhibitors

Jashmini Deka[1#], Aditya Mojumdar[2#], Pietro Parisse[1], Silvia Onesti[2*] and Loredana Casalis[1*]

Supplementary Information

Supplementary Figure S1

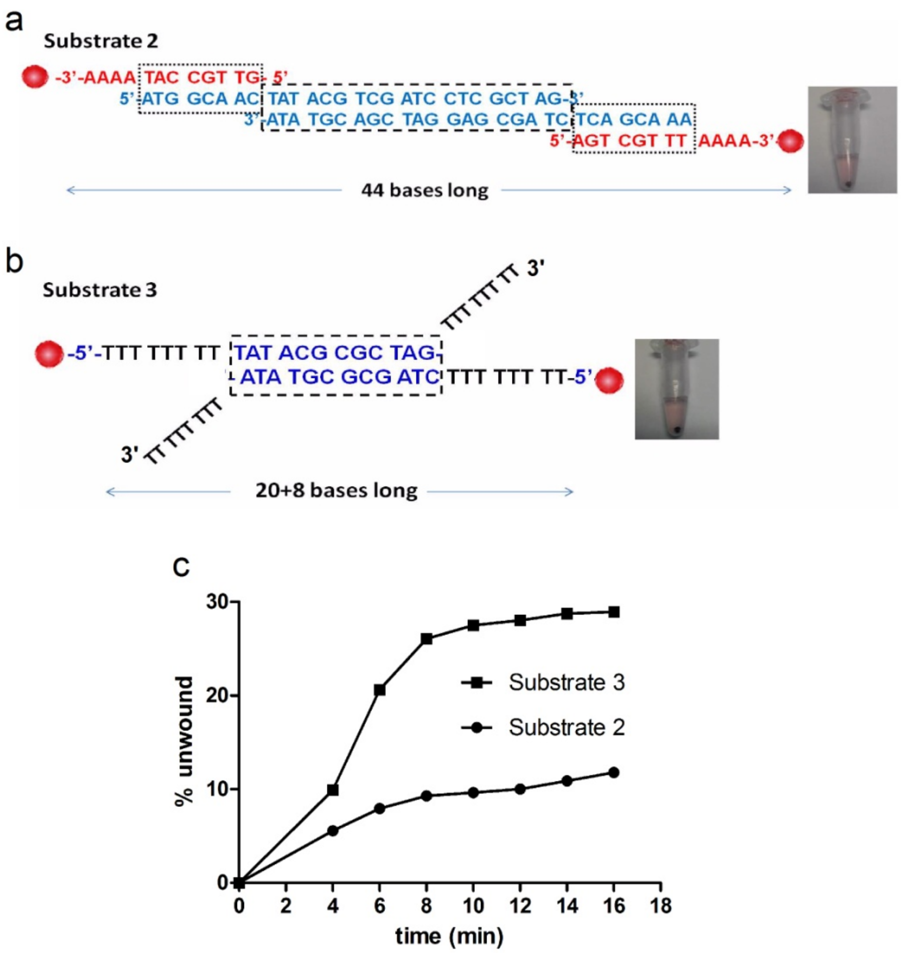

Supplementary Figure S1. (a) Schematic diagram of a longer blunt-ended duplex (Substrate 2). **(b)** Schematic diagram of a substrate with a 3' tail (Substrate 3). **(c)** DNA unwinding activity of the enzyme as a function of time for the two substrates.
